# Finger Amputation After Injection With Lidocaine and Epinephrine

**Published:** 2014-11-03

**Authors:** T. Ruiter, T. Harter, N. Miladore, A. Neafus, M. Kasdan

**Affiliations:** ^a^Department of Orthopedics, Western Michigan University Homer Stryker MD School of Medicine, Kalamazoo; ^b^Division of Plastic Surgery, University of Louisville, Ky; ^c^Surgery Service, Robley Rex VA Medical Center, Louisville, Ky

**Keywords:** epinephrine, amputation, necrosis, finger, lidocaine

**Figure:**
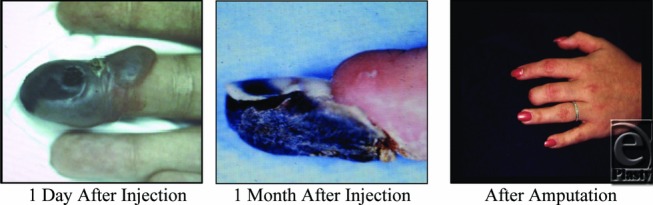


## DESCRIPTION

A 16-year-old previously well female, nonsmoker, without significant medical history, received an injection of lidocaine with epinephrine from her dermatologist, delivered in his office, to the radial and ulnar aspects of the middle phalanx of her long finger before having a skin lesion (wart) removed. No tourniquet was used. Eight hours later, the patient presented to the emergency department with acute pain of the involved long finger. Ischemic changes were noted on initial examination, and despite 1 week of intensive inpatient care, irreversible digital damage had occurred (Fig 1) and the patient went on to develop necrosis of the distal phalanx requiring amputation (Figs 1-3).

## QUESTIONS

1. **What is the effect of epinephrine on digital blood flow?**
2. **What are the benefits of using epinephrine with a local anesthetic?**
3. **May vasoconstriction due to local epinephrine injection be reversed?**
4. **What are the benefits of using plain lidocaine when anesthetizing a finger?**

## DISCUSSION

Hand surgeons routinely use lidocaine with epinephrine for digital anesthesia in elective and emergency hand surgical operations.^1^ We are unable to find another reported case of finger ischemia and amputation after injection of an epinephrine-containing local anesthetic. Digital ischemia after local anesthesia injection is thought to be of historic concern, owing to the previous use of procaine, which may deteriorate and result in a toxic drop in pH.^2^

When employed for digital anesthesia, epinephrine redistributes cutaneous blood and injected digits experience a low flow state, which is usually well tolerated. The temporary vasoconstriction allows for improved hemostasis, longer duration of analgesia, and decreases the need and risk of a tourniquet. The addition of epinephrine is known to potentiate and prolong the effect of analgesia, with fourfold greater duration being reported in the rat model.^3^

Epinephrine-induced vasoconstriction in the digit may be reversed through an alpha-blockade.^4^ Injecting phentolamine (1 mg/1 mL normal saline) directly into the compromised finger quickly reestablishes blood flow and decreases the likelihood of reperfusion pain or neuropraxia. Many reports of even high-dose epinephrine via auto injector (Epipen, Day Napa Calif) have been successfully managed without digital necrosis.^5^

The authors are uncertain if the epinephrine containing local anesthetic caused this patient's finger necrosis. The patient had no known prior medical (eg, Raynaud's) or psychiatric history, did not smoke or use illicit drugs, and gave no history of previous hand injury. Yet, it is possible that other events such as immersion in hot water, even if denied by the patient, could have possibly occurred. Also, reversal via phentolamine was not attempted. Knowledge of phentolamine as a rescue agent is important and may have saved the finger in this case if the true cause was indeed epinephrine vasoconstriction. However, our practice is to avoid epinephrine containing local anesthesia for use in the digits. We prefer plain marcaine or lidocaine with a tournicot as an alternative when anesthetizing fingers for surgery.
